# Robotic and Laparoscopic Inguinal Hernia Repair in Africa: Current Adoption, Challenges, and Future Horizons

**DOI:** 10.3389/jaws.2026.14775

**Published:** 2026-03-19

**Authors:** Adebayo Falola, Murtaja Satea, Pedro Vega Guillen, Rodolfo J. Oviedo

**Affiliations:** 1 University of Ibadan College of Medicine, Ibadan, Nigeria; 2 Department of Surgery, College of Medicine, University of Warith Al-Anbiyaa, Karbala, Iraq; 3 Universidad Peruana de Ciencias Aplicadas (UPC), Lima, Peru; 4 Department of Surgery, Nacogdoches Medical Center, Nacogdoches, TX, United States; 5 University of Houston Tilman J. Fertitta Family College of Medicine, Houston, TX, United States; 6 Sam Houston State University College of Osteopathic Medicine, Conroe, TX, United States

**Keywords:** inguinal hernia, laparoscopy, robotic surgery, Africa, LMICs

## Abstract

Inguinal hernia repair is one of the most common surgical procedures performed globally. Laparoscopic inguinal hernia repair (LIHR) is recognized globally to be effective and safe, with advantages over open surgery, but its implementation across the African continent has been slow, with only 12 countries reporting implementation, and only 3.3% of inguinal hernia repairs in sub-Saharan Africa performed using laparoscopic techniques. Robotic surgery, although still emerging within the continent, with around 20 robots primarily used in urology across South Africa, Egypt, Morocco, Angola, and Tunisia, no reports of robotic inguinal hernia repair currently exist. Progress, however, is being observed with the growing interest from surgical societies, private-sector robotic expansion, and humanitarian missions introducing mesh-based and limited laparoscopic procedures. Limitations include the low global utilization of minimally invasive surgery (MIS) for hernia repair despite guideline recommendations. This has been attributed to training challenges, steep learning curve, and limited evidence of benefit for bilateral and recurrent hernias. African-specific challenges include costs, inadequate training opportunities, surgeon preference, ongoing debates regarding its necessity in low-resource settings, lack of institutional support, and resource prioritization for other MIS procedures such as cholecystectomy and prostatectomy. Despite ongoing challenges, investments in research, training and cost-effective equipment, increased availability of mesh, and integration of humanitarian hernia missions into national training systems, can enhance adoption and contribute to better surgical outcomes for patients. This narrative review presents the present state of robotic and laparoscopic inguinal hernia repair in Africa, as well as the current challenges, and recommendations to improve adoption.

## Introduction

Inguinal hernia repair is a major surgical procedure worldwide, and due to the burden and feasibility of repair, the World Health Organization has categorized inguinal hernia as a “priority 1 surgical condition” [1, 2]. In Africa, the disease burden of inguinal hernias is high but only a fraction are treated, resulting in high rates of complications and mortality [3]. While there are about 175 cases per 100,000 people annually, only about 25 per 100,000 are treated [3, 4]. Minimally invasive surgery (MIS) techniques, including robotic and laparoscopic surgeries, have brought about several benefits to patients undergoing inguinal hernia repair by reducing postoperative pain, shortening hospital stays, complications, and facilitating quicker recovery [5, 6]. However, adoption of these new techniques in Africa remains low [7]. This is a review of the present status of robotic and laparoscopic inguinal hernia repair in Africa. A study of this nature has never been published in the English language, and this represents the first analysis of the current status of robotic and laparoscopic inguinal hernia repair in Africa with the goal of promoting a constructive discussion regarding challenges and opportunities for development in the future.

## Methodology

A comprehensive narrative review was conducted to map the current landscape of inguinal hernia repair in Africa. Electronic databases including PubMed, Google Scholar, and AJOL were searched for articles published up to December 2025. The search strategy combined terms for surgical techniques (“laparoscopy,” “robotic,” “TAPP,” “TEP”) with geographical descriptors (“Africa,” “Sub-Saharan,” and individual country names). The full search strategy is shown in Appendix 1. Observational studies and systematic reviews on MIS for inguinal hernia repair in Africa were considered eligible for review. The articles were screened, and data was extracted to present this review. References of included articles were also screened to ensure a coverage of grey literature and conference abstracts relevant to the region.

### Current Adoption of Laparoscopic Inguinal Hernia Repair in Africa

**FIGURE 1:**
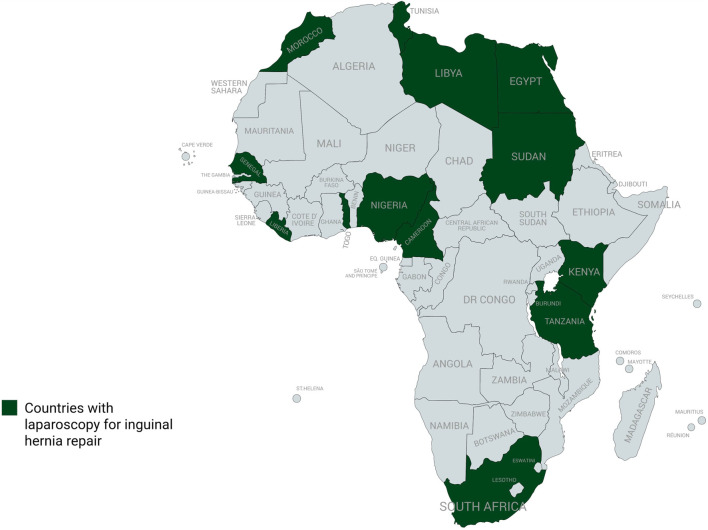
African countries where laparoscopy has been adopted for inguinal hernia repair.

Laparoscopic inguinal hernia repair (LIHR) has been extensively researched globally, and is now regarded as safe and efficient [8, 9]. The adoption of LIHR in Africa is increasing gradually, albeit more slowly compared to other settings [7, 10]. A retrospective audit conducted at a facility in South Africa demonstrated the viability and short-term efficacy of LIHR with potential for broader use on the continent [11]. A study focusing on adult groin hernia surgery in sub-Saharan Africa revealed that only 3.3% (750) of 23,657 procedures reviewed were performed using the Transabdominal Preperitoneal (TAPP) or Total Extraperitoneal (TEP) laparoscopic techniques [7]. Mesh fixation devices, trocars, laparoscopic suturing devices and other necessary equipment may be challenging for low-income facilities to procure [12]. Other barriers are lack of training opportunities and infrastructural constraints. On the other hand, Bassini, an open pure tissue technique, has been found to be predominant in sub-Saharan Africa because it is cost-effective and easy to learn [7]. A total of 12 countries, shown in Figure 1, have reported LIHR [7, 10, 11, 13–20], although the overall number of procedures performed is low compared to other settings [7, 10]. While the early practice of LIHR in Africa was reported in the late 1990s, there was only little progress until 2010 when the majority of reports began to come to light [10]. A systematic review which assessed the safety and effectiveness of LIHR in Africa reported only 2,329 procedures [10]. Another study recorded that only 1.54% (98/6381) of the laparoscopic general surgical procedures reviewed were performed for inguinal hernia repair [21]. The findings in these African-based studies are comparable to a recent global cohort report that revealed that as of 2024, access to minimally invasive repair was estimated at 12.5% in low- and middle-income countries (LMICs), and 1.1% in low-income countries (LICs) [22].

### Current Status of Robotic Inguinal Hernia Repair in Africa

Robotic-assisted surgery represents a progression in MIS, offering advantages over laparoscopy like enhanced dexterity, three-dimensional visualization, and ergonomic benefits for surgeons [23]. Although some LMICs in other regions have begun utilizing robotic surgery for inguinal hernia repair, this has not yet been reported in Africa [24–26]. In Asia, countries like India and Pakistan have reported adoption [24, 25]. Presently, there are approximately 20 surgical robots in Africa being used in various surgical specialties [26, 27]. A total of 12 of these robots are available in private healthcare facilities in South Africa [26–30], and two are in private institutions in Angola and Morocco [31, 32]. The remaining six are in use in public hospitals in Egypt, Tunisia, and Morocco [26, 27, 32]. These surgical robots are primarily used for urological procedures especially prostatectomy, while implementation in other areas such as abdominal wall surgery is still relatively developing [26].

## Discussion

### Challenges Hindering the Adoption of Minimally Invasive Techniques for Hernia Repair in Africa

There is generally low use of MIS for hernia repair globally regardless of income group, although lower in LMICs [22]. This is despite international guidelines recommending MIS when expertise and equipment is available [22, 33]. Its use is limited because of challenges such as technical difficulty, steep learning curve for surgeons, alongside limited access to specialized training. There is also a lack of evidence regarding clinical benefit for patients with bilateral and recurrent hernias [33]. The high costs of laparoscopic and robotic equipment, and shortage of skilled surgeons proficient in MIS are other notable barriers in resource-constrained settings such as in Africa [6]. The need to optimize resources may explain the wider adoption of laparoscopy for other less demanding and higher volume procedures like cholecystectomy and appendectomy, compared to hernia repair [21]. In addition, perceptions by surgeons and patients regarding LIHR affect its adoption [34]. Surgeons who are primarily used to open techniques may be hesitant to adopt laparoscopic methods which require additional training, especially when institutional support is not available [34]. Additionally, there are debates regarding the most appropriate method of repair, and whether the MIS is necessary for low-resource settings [35].

### Present Progress and Innovations

There are recent initiatives across Africa, which may be used as an inspiration in the new efforts to achieve an enhanced adoption rate of MIS to repair inguinal hernias. For example, training programs facilitated by international and local organizations have offered hands-on training to surgical trainees in Uganda and Nigeria [36]. In Rwanda, the establishment of “Institute for Research into Cancer of the Digestive System (IRCAD) Africa”, an international training center with minimally invasive and simulation technologies, has provided training opportunities for surgeons [27, 37]. Likewise, the Society of Endoscopic Surgery in South Africa runs laparoscopic hernia fellowships and maintains national guidelines and a hernia registry [38]. The increased availability of robotic equipment in the private sector in South Africa, Morocco, and Angola can also serve as inspiration for further private sector involvement across the continent [30–32]. Several surgical congresses are increasingly featuring sessions on robotic hernia repair, indicating growing interest and suggesting that the first reports will likely emerge soon [39, 40]. These include the Egyptian Society of Laparoscopic Surgery [39], the South African Society of Endoscopic Surgeons [40], the Algerian Society of Wall Surgery [41], and the Moroccan Society of Surgery [42]. Volunteer teams (humanitarian missions) have filled a gap in the provision of treatment services for inguinal hernia repair in Africa [43, 44]. Examples are “Operation Hernia” founded by the United Kingdom, and “Surgeons for Africa” founded by Germany, which have delivered high-volume open mesh hernioplasties in Ghana and Rwanda [43]. In Nigeria, missions have contributed to reduced local burden of inguinal hernia [45], and organizations like “GEANCO” Foundation have introduced laparoscopic procedures in their expanded surgical missions [44]. In certain African countries, private and tertiary hospitals have begun expanding their services to provide LIHR [37, 46–48]. For example, LIHR, is taught as a novel approach in tertiary facilities in South Africa [37]. Furthermore, initiatives to expand training access for MIS, such as partnerships with global surgery societies, offer potential for broader applications in the future [48]. There are also indications of gradual progress in robotic surgery’s contribution to surgical care in Africa. Countries such as South Africa and Egypt have taken early steps toward integrating robotic systems into their surgical practice, with growing interest in exploring its application beyond urology [26]. While high costs remain a barrier, the landscape of robotic surgery is evolving with the introduction of new, more cost-effective ‘modular’ robotic systems (such as the Hugo™ RAS system and CMR Versius) [49]. Unlike the traditional multi-port systems that require large dedicated operating theaters, these open-console, modular platforms offer flexibility and lower operational costs, making them potentially more suitable for resource-constrained settings in Africa [24]. The recent introduction of such systems in tertiary centers in developing regions suggests a shift towards more accessible robotic architecture.

### Pathways to Further Progress

To enhance the adoption of laparoscopic and robotic inguinal hernia repairs in Africa, several strategies can be considered. Conducting research to obtain the outcomes and cost-effectiveness of minimally invasive hernia repairs in Africa, compared with open techniques is important to inform policy decisions and encourage adoption [48]. Open non-mesh repair has been found to be the prevalent surgical technique in sub-Saharan Africa, accounting for up to 65% (15,510/23,657) of all inguinal hernia repair [7]. International guidelines and several global hernia societies have recommended the mesh for inguinal hernia repair because it reduces recurrence, and it is cost-effective [33]. Efforts to increase the availability of mesh for all patients undergoing inguinal hernia repair in Africa, as well as training programs for mesh techniques should therefore be the first step towards expanding access to surgical advances and technologies [22]. Incorporating basic laparoscopy training into institutional surgical training programs can help empower surgical trainees with both theoretical knowledge and practical surgical skills [50]. Partnerships with international institutions and organizations can alleviate financial burdens of the initial implementation costs in order to facilitate adoption [51]. Practical steps to implement training in robotic hernia repair have been modeled by the European Hernia Society (EHS) [52]. This involves simulation, “train-the-trainer”, and cadaveric robotic hernia courses. For implementation, regional training hubs should be created in national teaching hospitals or academic centers, and a validated stepwise curriculum should be adopted. The EHS pathway is an example of such a curriculum (pre-course e-learning → simulator proficiency benchmarks → cadaver or animal labs → supervised or proctorship practice → independent practice) [52]. Regarding the status of robotic surgery certificates, there is currently no unified, continent-wide credentialing framework in Africa, and data on local certification standards are scarce. To address this, African surgical societies can leverage the experience of established systems, such as in the United States, where the Society of American Gastrointestinal and Endoscopic Surgeons (SAGES) implemented consensus recommendations for robotic credentialing [53]. Therefore, adopting a similar curriculum-based certification model in Africa is recommended to facilitate the safe and effective integration of robotic surgery. Telesurgery and remote mentoring can be used with hands-on sessions to deliver training courses. The IRCAD online courses, for example use such hybrid formats [54, 55]. For telesurgery and remote proctorship to be viable, investment in digital infrastructure is a prerequisite. The latency requirements for safe robotic telesurgery necessitate stable high-speed internet (e.g., 5G or fiber optics) [55]. Therefore, advocacy for surgical robotics in Africa must go hand-in-hand with national initiatives to improve digital connectivity, ensuring that remote training hubs in South Africa, Egypt, or Rwanda can effectively mentor surgeons in remote locations without signal latency compromising patient safety. For sustainability purposes, “train-the-trainer” activities should be used to create local faculty and reduce dependence. The humanitarian hernia missions mentioned earlier can also be leveraged towards capacity building for minimally invasive inguinal hernia repairs through collaborations with tertiary institutions and health ministries [43, 56]. Missions can implement “train-the-trainer” courses, which will allow visiting surgeons to provide practical guidance and training to local surgeons, and also facilitate the donation of MIS equipment through strategic partnerships with equipment manufacturers [56]. A summary of the challenges hindering adoption, present progress and innovations, and pathways to further progress is shown in Table 1.

**TABLE 1:** Summary of challenges, progress, and pathways to enhance adoption of minimally invasive hernia repair in Africa.

Category	Summary
Challenges hindering adoption	Low global utilization of MIS for hernia repair despite guideline recommendations
	Technical difficulty and steep learning curve for laparoscopic and robotic hernia repair
	Limited access to specialized training and few skilled surgeons proficient in MIS
	Limited evidence of clinical benefit for bilateral and recurrent hernias
	High costs of laparoscopic instruments, meshes, and robotic systems
	Resource intensity of LIHR compared with higher-volume MIS procedures (e.g., cholecystectomy, appendectomy)
	Surgeon and patient perceptions: hesitancy toward MIS, and preference for familiar open techniques
	Institutional barriers: lack of support, limited training programs
	Debates about suitability of MIS for low-resource settings
Present progress and innovations	Hands-on training programs in Rwanda, Uganda, and Nigeria, supported by international and local organizations
	Establishment of IRCAD Africa (Rwanda) providing state-of-the-art MIS and simulation training
	Society of endoscopic surgery in South Africa offering laparoscopic hernia fellowships, national guidelines, and a hernia registry
	Growing private-sector investment in robotic systems
	Increasing adoption of laparoscopic hernia repair in private and tertiary centers across Africa
	Humanitarian hernia missions providing high-volume open repairs, skills exposure, and beginning to introduce MIS techniques including laparoscopy
Pathways to Accelerate progress	Conduct outcome and cost-effectiveness research comparing MIS vs. open hernia repair to guide policy
	Increase access to mesh and mesh-based training for open and MIS repairs
	Integrate basic laparoscopy training into surgical residency curricula
	Partnerships with international bodies to reduce financial strain and support early implementation
	Adopt structured robotic hernia training, modeled after EHS curricula
	Develop regional training hubs (e.g., IRCAD Africa; national teaching hospitals)
	Expand remote learning using telesurgery, tele-mentoring, and hybrid courses
	Establish train-the-trainer programs to build sustainable local centers

### Limitations

This review has certain limitations. While the authorship is expanded to include a multi-national perspective (Nigeria, USA, Iraq, and Peru), the primary African context relies heavily on data and experiences from West Africa (specifically Nigeria), a lower-middle-income setting. Consequently, the interpretation of adoption patterns may differ from the personal experiences of surgeons in upper-middle-income African nations, such as South Africa or Algeria, where resources and infrastructure are comparatively advanced. Future reviews involving a broader consortium of African surgeons from diverse economic regions would be beneficial to provide a more holistic pan-African perspective.

## Conclusion

The adoption of laparoscopy for inguinal hernia repair in Africa faces challenges, and the application of robotic surgery for hernia repair is yet to be reported. Alongside continued investments in training, research and healthcare infrastructure, the present signs of progress indicate wider future use of surgical advances in the management of hernia in Africa. There is ample opportunity to promote surgical education and collaboration in the African continent to use the local talent while enabling its surgeons to have access to technological advances and serve their communities and patients.

## Appendix 1: Search Strategy

(robotic OR robot-assisted OR robot OR laparoscopy OR laparoscopic OR “transabdominal preperitoneal” OR “totally extraperitoneal” OR TAPP OR TEP) AND (Hernia OR “inguinal hernia” OR “groin hernia”) AND (“surgery” OR “procedure”) AND (Africa OR Algeria OR Angola OR Benin OR Botswana OR “Burkina Faso” OR Burundi OR “Cabo Verde” OR Cameroon OR “Central African Republic” OR Chad OR Comoros OR Congo OR Brazzaville OR Kinshasa OR “Cote d’Ivoire” OR “Ivory Coast” OR Djibouti OR Egypt OR “Equatorial Guinea” OR Eritrea OR Eswatini OR Ethiopia OR Gabon OR Gambia OR Ghana OR Guinea OR Guinea-Bissau OR Kenya OR Lesotho OR Liberia OR Libya OR Madagascar OR Malawi OR Mali OR Mauritania OR Mauritius OR Morocco OR Mozambique OR Namibia OR Niger OR Nigeria OR Rwanda OR “Sao Tome and Principe” OR Senegal OR Seychelles OR “Sierra Leone” OR Somalia OR “South Africa” OR “South Sudan” OR Sudan OR Tanzania OR Togo OR Tunisia OR Uganda OR Zambia OR Zimbabwe).
